# Central Neurocytoma in the Fourth Ventricle: A Case Report and Review of Cases With This Unusual Presentation Site

**DOI:** 10.7759/cureus.96245

**Published:** 2025-11-06

**Authors:** Esli Dimayuga-Velazquez, Leoncio Tovar-Romero, Wilson Toscano-Rengifo, Aurea Escobar-España, Luis Adrian Tellez-Manriquez, Erick Gomez-Apo

**Affiliations:** 1 Neurological Surgery, Hospital General de México, Mexico City, MEX; 2 Pathology, Clínica Hospital Axxis, Quito, ECU; 3 Pathology and Neuropathology, Hospital General de México, Mexico City, MEX; 4 Medicine, National Autonomous University of Mexico, Mexico City, MEX

**Keywords:** central neurocytoma, fourth ventricle, histopathology, neuronal markers, unusual site

## Abstract

Central neurocytoma (CN) is classified as a grade 2 neuronal tumor and is mostly located in the lateral ventricles. In this report, we present the case of a 40-year-old female patient who was admitted due to vertigo and a holocranial headache. Magnetic resonance imaging (MRI) revealed a lesion in the fourth ventricle that appeared heterogeneous and hyperintense. In the T2/fluid attenuated inversion recovery (FLAIR) sequence, numerous cystic areas were observed. Light microscopic examination showed a neoplasm with a neuronal phenotype; areas with neoplastic cells exhibiting a ganglionic appearance were noted, along with immunohistochemical expression of neuronal markers. Currently, the patient is showing clinical improvement.

## Introduction

Central neurocytoma (CN) is a rare tumor of the central nervous system (CNS) with an incidence of 0.1%-0.5% of all types of primary brain neoplasms [1]. In the first two editions of the WHO classification of CNS tumors (1979 and 1993), the group of neuronal tumors is described, but CN was not mentioned [2, 3]. This neoplasm was first described by Hassoun J et al. (1982), who reported two adult patients with a neoplasm originating from the fornix [4].

In the third edition of the WHO classification of CNS tumors (2000), CN was described in the international classification for the first time in the group of neuronal and mixed neuronal-glial tumors, and grade II was assigned [5]. The fourth edition of the WHO classification of CNS tumors (2007) describes CN and adds extraventricular neurocytoma, in addition to including "atypical neurocytoma" in cases with expression of Ki-67 indices greater than 2% [6].

In the revision of the fourth edition of the WHO classification of CNS tumors (2016), there are generally no notable changes [7]. In the fifth edition of the WHO classification of CNS tumors (2021), the CN corresponds histologically to grade 2. The proliferation index is considered a powerful prognostic marker, but this index for predicting prognosis is still under debate [8]. They are common in young adults and usually have an excellent prognosis after surgical resection. Typical clinical manifestations include symptoms of increased intracranial pressure, although no clinical feature is pathognomonic to CN [9]. CN was initially conceptualized as a tumor of neural origin, as the pathology demonstrated morphology and expression patterns consistent with neurons [10].

Traditionally, there have been two hypotheses about the origin of CN. One hypothesis suggests that the CN originates from the septum pellucidum, and the second hypothesis suggests that the CN originates from remnants of the subependymal germinal area of the lateral ventricle [11]. Most CNs are found in the anterior half of the lateral ventricle, although some have been reported to be found in the third and fourth ventricles. The tumor is also usually attached to the septum pellucidum near the foramen of Monro [12]. Various extraventricular locations have been reported, such as in the cerebral hemispheres, thalami, amygdala, sellar region, pons, cerebellum, and spinal cord [13].

The diagnosis of CN should be considered an entity within intraventricular lesions. The evaluation should include non-contrast CT, magnetic resonance imaging (MRI) with and without gadolinium contrast, and magnetic resonance spectroscopy. CN classically presents as a tumor arising from the septum pellucidum or foramen of Monro, has a partially calcified area on CT, with a soap-bubble multicystic appearance on MR T2-imaging, and heterogeneous enhancement on T1-weighted post-gadolinium MRI [14]. By spectroscopy, CN is characterized by high levels of choline and glycine, low levels of NAA, and absent or low levels of Ala compared to intraventricular meningioma. The choline/creatinine and NAA/creatinine ratios seem to be significantly higher in CN compared to other intraventricular tumors [15]. CN has some distinctive features on MRI, such as the “scalloping sign”, “broad-based attachment”, and “gemstone enhancement” [15].

Histopathologically, CN can be heterogeneous, displaying areas that vary within the same sample. This may include a "honeycomb pattern" and the presence of small cells with scant cytoplasm and granular chromatin. Morphologically, CN may exhibit features similar to oligodendroglioma, and it can also present perivascular rosettes similar to those found in ependymoma, which raises the potential for misdiagnosis [12]. Because CN is histopathologically similar to other brain neoplasms, immunohistochemistry provides the definitive diagnosis. By immunohistochemistry, CN is positive for synaptophysin, positive for neuron-specific enolase, and negative for glial fibrillary acidic protein [16].

Maximal resection is currently considered the ideal therapeutic option, as well as the best long-term prognostic factor. Adjuvant radiotherapy appears to benefit patients with incomplete resection and "atypical neurocytoma" [17].

## Case presentation

The patient was a 40-year-old female. Her current condition began a year ago with vertigo; subsequently, a holocranial headache of intensity 7/10 on a Visual Analog Scale (VAS) of the oppressive type, which partially subsides with the intake of analgesics, was added; in addition, she presented intermittent ataxia, nausea, and occasional emesis, leaving it to evolve freely. The clinical manifestations correspond to the data of obstructive hydrocephalus. She presented with drowsiness and generalized onset seizures, for which reason she was admitted to our hospital for a diagnostic workup. 

On physical examination, the patient was found to have drowsiness, horizontal nystagmus predominantly on the left, bilateral papilledema, preserved facial symmetry, generalized muscle strength and sensitivity, ataxic gait, and bilateral dysmetria.

**Figure 1:**
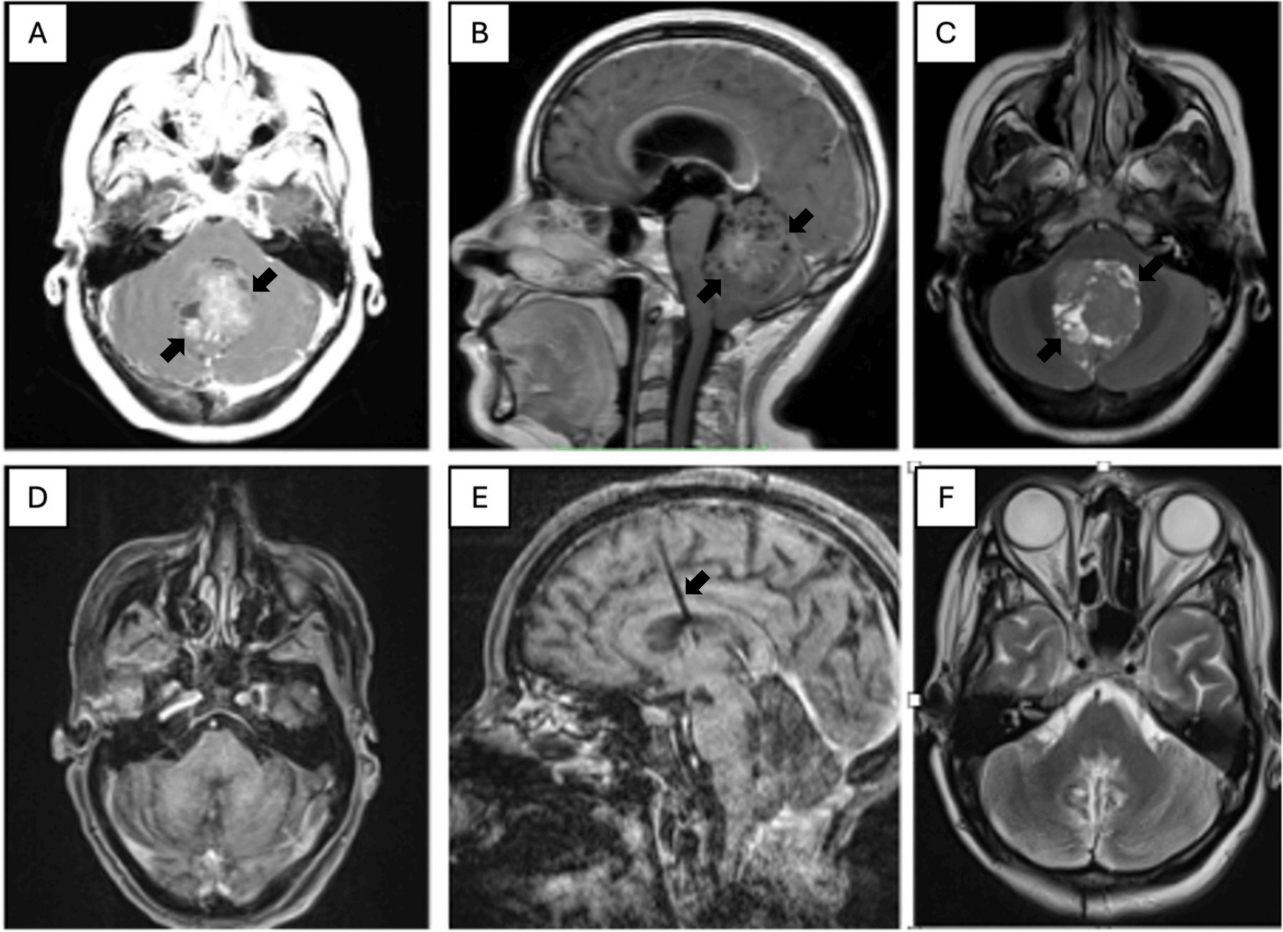
Magnetic resonance imagining reports A. Axial in contrast-enhanced T1 sequence showing a lesion dependent on the fourth ventricle (arrows) with heterogeneous enhancement to the contrast medium. B. Sagittal in contrast-enhanced T1 sequence revealing a lesion dependent on the roof of the fourth ventricle (arrows), with heterogeneous enhancement to the contrast medium, and collapse of the ventricular system with evidence of lateral ventricle dilation. C. Axial in T2 sequence showing a lesion with heterogeneous solid and cystic areas, with probable calcifications inside (arrows). D. Axial in contrast-enhanced T1 sequence demonstrating total resection of the lesion, with adequate expansion of the cerebellar hemispheres. E. Sagittal in contrast-enhanced T1 sequence showing the fourth ventricle without evidence of neoplastic lesion, with postsurgical changes secondary to resection, and a precoronal ventriculoperitoneal shunt (arrow), with no signs of hydrocephalus. F. Axial in T2 sequence showing no evidence of residual lesion, with no cerebellar or brainstem lesion detected.

A contrast-enhanced MRI of the brain was performed, which identified a lesion located in the fourth ventricle, which was heterogeneous and hyperintense to contrast medium in contrast-enhanced T1; in the T2/fluid attenuated inversion recovery (FLAIR) sequence, there were numerous cystic areas, with restriction to diffusion in its solid components and the presence of calcifications inside, causing compression of the fourth ventricle and hydrocephalus (Figure 1).

A high-pressure ventriculoperitoneal shunt system was placed, and the lesion was subsequently resected by middle suboccipital craniectomy and resection of the posterior arch of C1, with a paravermian telovelar approach, identifying a lesion dependent on the roof of the fourth ventricle, whitish gray, soft consistency, and slightly vascularized.

**Figure 2:**
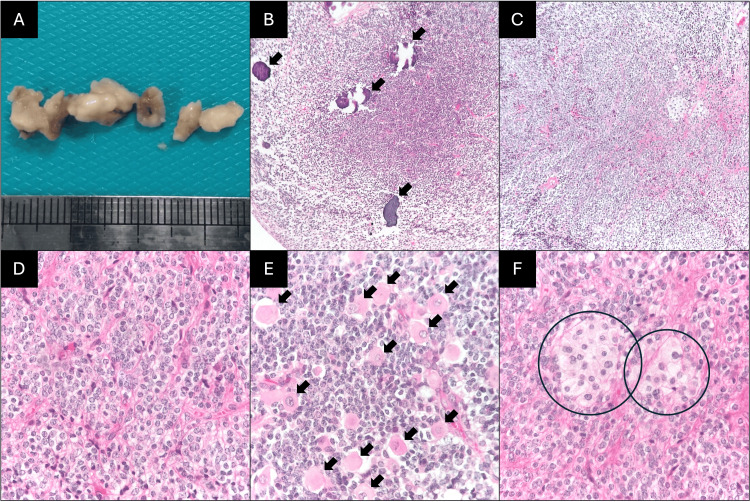
Histopathology reports A. Gross appearance of the obtained tissue fragments. B. Neoplasm exhibiting calcifications (arrows) (H&E, 40X). C. Panoramic view showing solid growth of neoplastic cells (H&E, 40X). D. Cytological detail: the cells are medium-sized, with oval nuclei, granular chromatin, eosinophilic staining, and limited cytoplasm vascularized by capillaries (H&E, 400X). E, F. Detailed areas showing neuronal ganglion differentiation (arrows and circles) (H&E, 400X).

In the histological sections examined, a neoplasm with a neuronal phenotype with a solid growth pattern, vascularized by thin capillaries and with focal calcium deposits, was observed; the neoplastic cells were medium-sized, with an oval nucleus, granular chromatin, eosinophilic and scarce cytoplasm, and the chromatin had a salt and pepper appearance. The atypia was minimal, with an absence of mitosis and an absence of necrosis. In some areas, neoplastic cells with a ganglionic appearance were identified, characterized by nuclei with similar characteristics and with eosinophilic and dense cytoplasm, and no residual tissue was evident (Figure 2).

**Figure 3:**
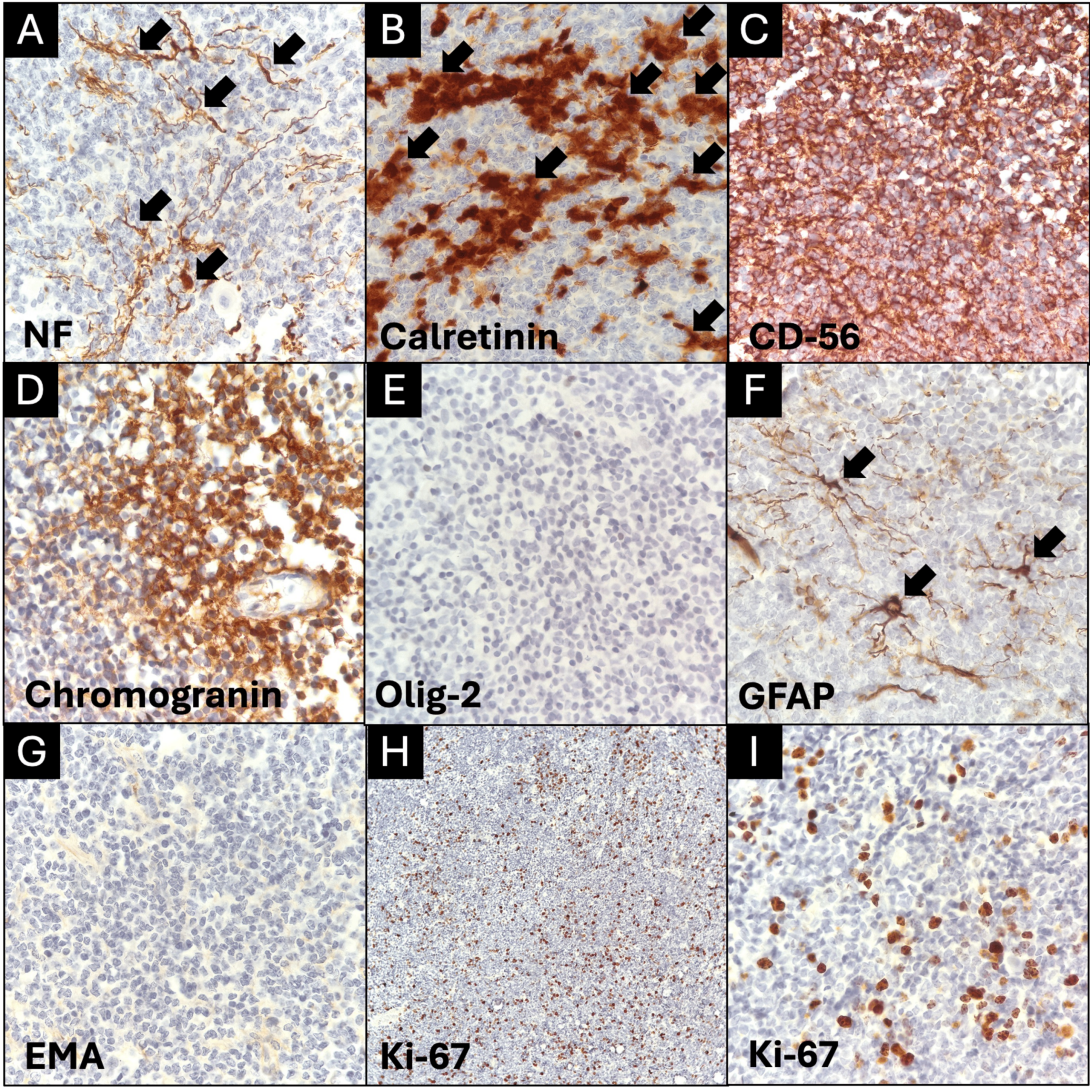
Immunohistochemistry reports A. Neurofilament antibody (100X), focally positive in neoplastic cells (arrows). B. Calretin (100X), positive in neoplastic cells (arrows). C. CD-56 (100X), positive in neoplastic cells. D. Chromogranin (100X), positive in neoplastic cells. E. Olig-2 (100X), negative in neoplastic cells. F. GFAP (100X), negative in neoplastic cells; positive expression in reactive astrocytes (arrows). G. EMA (100X), negative in neoplastic cells. H. Ki-67 (40X), positive in neoplastic cells (30%). I. Ki-67 (400X), positive in neoplastic cells (30%).

Immunohistochemical reactions were performed, with positive expression for neurofilaments, calretinin, CD-56, and chromogranin antibodies; the cells were negative for Olig-2, GFAP, and epithelial membrane antigen antibodies; and the cell proliferation index (Ki-67) had positive expression in 30% of the neoplastic cells (Figure 3, Table 1).

**Table 1:** Antibody test reports

Antibody	Company	City	Country	Dilution
Neurofilaments	Diagnostic BioSystems Inc.	Pleasanton, CA	United States	1:800
Calretinin	Biocare Medical	Pacheco, CA	United States	1:100
CD-56	Biocare Medical	Pacheco, CA	United States	1:100
Chromogranin	Agilent Dako	Santa Clara, CA	United States	1:200
Olig-2	ZETA Corporation	Sierra Madre, CA	United States	1:200
GFAP	Diagnostic BioSystems Inc.	Santa Barbara, CA	United States	1:800
EMA	Biocare Medical	Pacheco, CA	United States	1:100
Ki-67	Biocare Medical	Pacheco, CA	United States	1:100

At the follow-up visit, the patient was awake, with a 15-point Glasgow Coma Scale score; pupils were 3 mm, isochoric, and normoreactive. She presented with right eye nystagmus and left peripheral facial paralysis. The remaining cranial nerves were unremarkable. Muscle strength is 4-/5 in all four limbs. She had generalized hyperreflexia, with bilateral Hoffmann, Trömner, and Babinski signs. There was no evidence of meningeal irritation.

## Discussion

The presentation of CN in the fourth ventricle is uncommon, and its imaging characteristics resemble those observed in the third ventricle and lateral ventricles. To our knowledge, 12 cases have been reported to date in the literature, plus the current case report [18-29]. Among these cases, six patients were male and seven were female. The symptoms varied widely and included ataxia, blurred vision, headache, nystagmus, vomiting, diplopia, gait disturbances, and loss of consciousness. The cell proliferation index (Ki-67) ranged from 1% to 50% (Table 2).

**Table 2:** Review of literature of CN in fourth ventricle (12 cases and current case) CN: central neurocytoma; M: male; F: female

Author	Age (years)	Sex	Presenting complaint	Imaging	Surgery	Ki-67 score
Warmuth-Metz et al. (1999) [18]	17	M	Ataxia and blurring of vision	Homogenous enhancement, calcification, and hydrocephalus	Partial resection + radiotherapy	10%
Hsu et al. (2002) [19]	35	M	Headache and blurring of vision	Heterogeneously enhanced, cystic	Gross total resection	2–10%
Cook et al. (2004) [20]	58	F	Headache, ataxia, and nystagmus	Heterogeneous enhancement, calcification	Partial resection + radiotherapy	<1%
Gallina et al. (2005) [21]	68	F	Headache and vomiting	Heterogeneously enhanced, calcification, and hemorrhage	Gross total resection	1%
Cultrera et al. (2005) [22]	28	M	Diplopia and gait disturbances	Homogeneous enhancement	Gross total resection	6%
Böhm et al. (2006) [23]	8	M	Diplopia and gait disturbance	Homogeneous enhancement, calcification	Incomplete resection + radiotherapy + chemotherapy (acute lymphatic leukemia)	50%
Ho et al. (2010) [24]	30	F	Headache	Heterogeneously enhanced, hemorrhage	Excised through a suboccipital approach	<2%
Jain et al. (2012) [25]	7	M	Headache and vomiting	Heterogeneous enhancement, hemorrhage, cystic	Gross total resection + radiotherapy	10–20%
Richardson et al. (2019) [26]	70	F	Nausea, vomiting, and vertigo	Heterogeneous enhancement	Near total excision	5–7%
Rai et al. (2019) [27]	8	F	Headache and blurring of vision	Intraventricular tumor arising from the floor of the fourth ventricle	Gross total resection	1%
Ferrigno et al. (2020) [28]	48	M	Frontal headache	Inferior third ventricle tumor, cerebral aqueduct, and expansion into the fourth ventricle	Transcallosal resection + radiotherapy	3-4%
Unal et al. (2024) [29]	43	F	Losing consciousness	Left cerebellar lesion with ventricular hemorrhage	Gross total resection	9%
Dimayuga-Velazquez et al. (current case)	40	F	Headache, ataxia, nausea, and vomiting	Homogenous enhancement, calcification	Gross total resection	30%

Intraventricular tumors account for 2% of intracranial lesions, and fourth ventricular lesions represent only a fraction of this subset [30]. Tumors of the IV ventricle represent 1%-5% of all intracranial lesions. The most common tumor types in the IV ventricle and brainstem are medulloblastomas (7%-93.3%), metastases (4.8%-46.4%), ependymomas (6.7%-38%), astrocytomas (grade 1-4) (7.3%-33%), subependymomas (9%-19.5%), choroid papillomas (2.2%-14.6%), and hemangioblastomas (4.9%-14.3%) [31]. The cellular origin of CN is unknown. Evidence of both glial and neuronal differentiation in some tumors suggests an origin from neuroglial precursor cells with the potentiality of dual differentiation. CNs could originate from the subependymal plate of the lateral ventricles [8]. However, an origin from the circumventricular organs has also been proposed. Its localization in the area postrema region led to the hypothesis that it may be derived from this circumventricular organ [32]. Histologically, the diagnosis was challenging because the morphology resembled ependymoma. Fortunately, in this case, there was sufficient material, areas of ganglionar differentiation were found, and immunohistochemistry was aimed at confirming the diagnosis of CN and ruling out the small possibility of ependymoma.

The Ki-67% is clearly elevated, but the current classification does not assign a higher histological grade. However, due to this, the Department of Neurosurgery will conduct longer follow-up.

## Conclusions

When diagnosing CN of the fourth ventricle, it should be considered only as a last option. Special attention must be given to distinguishing it from choroid plexus tumors and ependymomas, which are much more common in this localization. The histopathological morphology of the lesion resembles some characteristics typically seen in grade 2 ependymomas. Notable features indicating neurocytoma include the presence of calcifications, a solid growth pattern, and the maturation of neoplastic cells into ganglion-like neuronal cells.

Because of these similarities, immunohistochemistry is performed to differentiate between neurocytoma and ependymoma, with results ultimately supporting the diagnosis of neurocytoma. In the fields of neurosurgery, neuroimaging, and neuropathology, we often encounter unusual cases like this one. Effective communication among the various disciplines within neuroscience is essential for achieving accurate diagnoses and providing appropriate treatment for patients.
